# Evaluation the Effects of Some Relevant Parameters on Elastic Modulus of Pumpkin Seed and Its Kernel

**DOI:** 10.1155/2012/271650

**Published:** 2012-02-21

**Authors:** Mohammad Hossein Abbaspour-Fard, Rasool Khodabakhshian, Bagher Emadi, Hasan Sadrnia

**Affiliations:** Department of Agricultural Machinery Engineering, Ferdowsi University of Mashhad, P.O. Box 91775-1163, Mashhad, Iran

## Abstract

The elastic modulus of two varieties of Iranian pumpkin seed and its kernel (namely, Zaria and Gaboor) were evaluated as a function of size (large, medium, and small), loading rate (2, 5, 8, and 10 mm/min), and moisture content (4, 7.8, 14, and 20% d.b) under quasistatic compression loading. The results showed that elastic modulus of pumpkin seed and its kernel decreased with increasing moisture content and also increasing loading rate, for the varieties under study. The average modulus of elasticity of pumpkin seed from 68.86 to 46.65 Mpa and from 97.14 to 74.93 Mpa was obtained for moisture levels ranging from 4 to 20%, for Zaria and Gaboor varieties, respectively. The elastic modulus of pumpkin seed decreased from 73.55 to 43.04 Mpa and from 101.83 to 71.32 Mpa with increasing loading rate from 2 to 10 mm/min for Zaria and Gaboor varieties, respectively.

## 1. Introduction

Pumpkin seed (*Cucurbita maxima*) is considered to be an important oilseed crop, because it contains highly nutritious oil in large quantity. According to Iranian government statistical data (2008), over 20 varieties of pumpkin are cultivated in Iran. The majority of pumpkin seed is consumed for oil production (85%) and the rest for fresh consumption as nuts. The oil of Pumpkin seed has strong antioxidant properties and several other health benefits such as hampering the growth and reduction of the size of prostate, retardation of hypertension, mitigation of hypercholesterolemia and arthritis, reduction of bladder and urethral pressure, improving bladder compliance, alleviation of diabetes by promoting hypoglycemic activity, and lowering the levels of gastric, breast, lung, and colorectal cancer [1, 2].

In Iran, the oil of pumpkin seeds is mostly obtained by mechanical extraction. However, this process suffers from some disadvantages. The presence of a justly high percentage of hulls in the seed not only causes rapid wear of the moving parts of the expeller but also reduces the total oil yield. The transfer of pigments from the hull to the extracted oils leads to high specific energy and yields cakes of lower food value. Therefore, pumpkin seeds should be hulled before entering the industrial process of oil extraction. Information of elastic characteristics of pumpkin seed*∖*kernel and their dependency on moisture content and size under compressive loading is essential for a rational design of an efficient dehulling system and equipment for mechanical expression of oil and other processes.

Owing to the complex shape of most of agricultural produces and their associated complex structure, the determination of a reliable elastic modulus presents a number of problems. For instance, one of the most important problems to determine the elastic modulus of agricultural produce is its viscoelastic characteristic, that is, it simultaneously behaves as elastic solid and viscous liquid. However, many researches have found that when small loads are applied in short times, these problems can fade, to a certain extent, using some methods based on elasticity theory [3–18].

The review of literature indicates that many studies have determined the elastic modulus of agricultural produce from force-deformation curves, based on Hertz theory [19]. Khazaei [15] studied the elastic modulus of pea pod, at loading rate of 5 mm/min and moisture content of 18% w.b. Kiani et al. [18] determined elastic modulus of red bean grains as a function of moisture content and loading rate. They reported that the Young's modulus of this grain decreased from 253.26 to 93.06 Mpa with increasing moisture content from 3% to 15%. Moreover, its young's modulus increased as loading rate increased from 3 to 15 mm/min.

However, there is no enough published work related to the effect of relevant parameters on elastic modulus of pumpkin seed and its kernel such as moisture content, size, loading rate, and variety. The present study aimed to investigate the effect of these parameters on elastic modulus of two major commercial Iranian varieties of pumpkin seed and its kernel, namely, Zaria and Gaboor.

## 2. Materials and Methods

### 2.1. Sample Preparation

**Figure 1:**
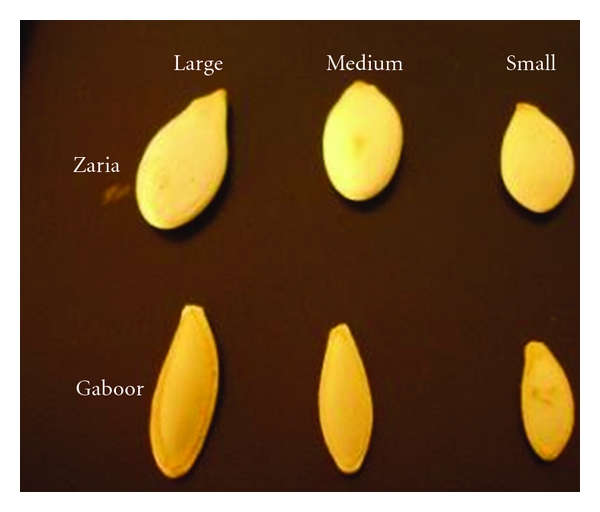
The size categories of two Iranian varieties of pumpkin seed.

Zaria and Gaboor varieties of pumpkin seed were obtained from different regions of Khorasan Razavi province (north east of Iran) during autumn season in 2010 (Figure 1). A mass of twenty kilograms from each variety of pumpkin was collected. At first, the pumpkin seeds were manually cleaned to get rid of foreign materials and broken and immature seeds. To prepare the samples of whole kernels, a part of the seeds equal to 10 kg was randomly separated and manually dehulled from each variety. The initial moisture content of seed and kernel samples was determined using the standard hot air oven method with a temperature setting of 105 ± 1°C for 24 h [20–23]. The initial moisture content of the seeds of Zaria and Gaboor varieties was 7.8% and 7.6% d.b, respectively. In the mean time, the moisture content of the corresponding kernels at this stage was 6.2% and 5.8% d.b., respectively. According to Khodabakhshian et al. [24], the seeds were sieved into three size categories (small, medium, and large) using 5.5, 6.5, and 8 mm square mesh sieves. The elastic modulus of pumpkin seed and its kernel was measured in four moisture content levels in the range of 4 to 20% (d.b.) that is a usual range since harvesting, transportation, storage, and processing operations of pumpkin seed. To provide the seeds and kernels with the desired moisture content, subsamples of both seeds and kernels of each variety and size category, each weighing 0.5 kg, were randomly drawn from the bulk sample and dried (oven method at 75°C for 2 h) or adding calculated quantity of water to the seeds, through mixing and then sealing in separate polyethylene bags of 90 *μ*m thickness. The samples were kept at 5°C in a refrigerator for 7 days to distribute the moisture uniformly throughout the sample. Before starting the tests, the required quantities of seed and kernel were taken out of frig and allowed to warm up to room temperature for approximately 2 h [20, 24, 25].

### 2.2. Elastic Modulus Measurement

**Figure 2:**
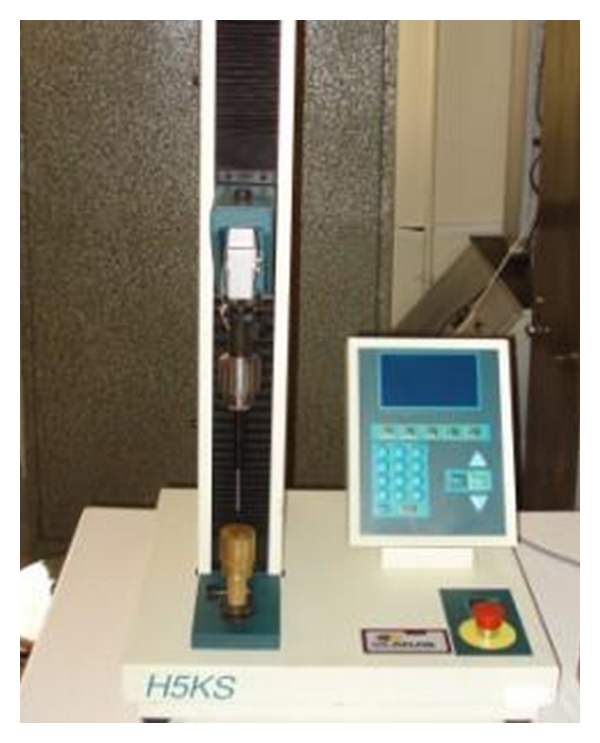
Universal test machine used in the compression test.

**Figure 3:**
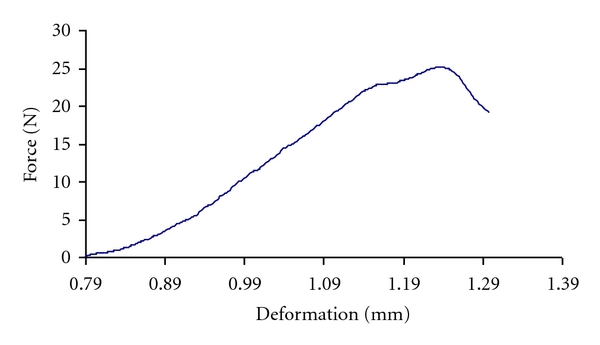
Typical force-deformation characteristics of pumpkin seed.

An Instron Universal Testing Machine (Model H5KS, Tinius Olsen Company) equipped with a 500 N compression load cell and integrator was used for the compression test of pumpkin seed and its kernel. Individual seeds or kernels were loaded between two parallel plates of the machine (Figure 2), compressed at the preset condition until rupture occurred as is denoted by a bioyield point in the force-deformation curve. A typical obtained force-deformation curve for a pumpkin seed is shown in Figure 3. As soon as the bio-yield point was detected, the loading was stopped. According to ASAE [26], 96 series of tests (two varieties: Zaria and Gaboor; four levels of moisture content: 4, 7.8, 14, and 20%; three size categories: small, medium, and large; four loading rates: 2, 5, 8, and 10 mm/min) were conducted. Data on the strength properties were automatically obtained from the integrator.

### 2.3. Statistical Analysis

The experiments were conducted with four replications for each moisture contents, varieties, size categories, loading rates, and the average values reported. Average, minimum, maximum, standard deviations, and regression equations were computed using Microsoft Excel software (2003). The analysis of variance (ANOVA) was carried out on a completely randomized design with factorial experiment using SPSS16 software. The *F* test was used to determine the significance of independent variables, and significant differences of means were compared using the Duncan's multiple ranges test at 5% significant level.

## 3. Results and Discussion

The variance analysis of the data indicated that all the studied variables namely moisture content, variety, size, and loading rate exhibited a significant effect on the modulus of elasticity for both seed and kernel of pumpkin (*P* < 0.99). Based on the statistical analyses, the interaction effect of moisture content × variety and loading rate × variety on the modulus of elasticity of pumpkin seed was not significant at 1% level, while created a significant effect on modulus of elasticity for kernel (*P* < 0.99). Also, the interaction effect between variety and size, loading rate and size, moisture content and size, and also between moisture content and loading rate was found to have significant effects (*P* < 0.99) on the modulus of elasticity for both seed and kernel of pumpkin. Variance analysis of data for seed also indicated that all three interaction effects of the variables with the exception of interaction effect of moisture content × size × loading rate were not significant. However, all three interaction effects of the variables showed significant effect (*P* < 0.99) on elastic modulus of pumpkin kernel. Moreover, variance analysis of data for both seed and kernel indicated an interaction effect at 1% level among variety, moisture content, size, and loading rate. In the following sections, the effects of each variable on the modulus of elasticity of pumpkin seed and its kernel are comprehensively discussed.

### 3.1. Size

**Figure 4:**
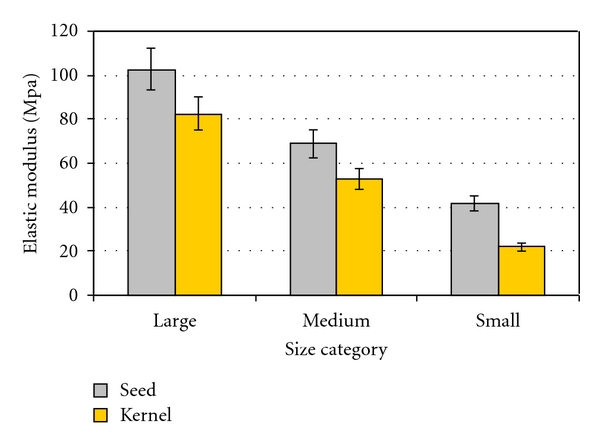
Effect of size on elastic modulus of pumpkin seed and its kernel.

Stepwise analysis of the obtained results revealed that among the studied variables including variety, moisture content, size category, and loading rate, the dominant factor on the elastic modulus of the seed and also kernel is the size. The variation of elastic modulus of seed and kernel at different size categories is shown in Figure 4. As it can be seen from this figure, the elastic modulus of pumpkin seed and its kernel increased as size increased from small to large. In other words, the average elastic modulus of large seeds was around 2.46 times more than that of small seeds. In the same way, the average elastic modulus of large kernels was about 3.77 times more than the small kernels. This trend can be related to the geometric mean diameter of sample (seed or kernel). Also, it can be attributed to the mass of the individual seed or kernel per unit volume with size category. On the other hand, the discrepancies between the elastic modulus of seeds and their kernels can be related to the cell structure and the variation of physical properties in seeds and kernels. This justification has been proved by Khodabakhsina et al. [24] on engineering properties of sunflower seed and its kernel. The increasing trend of elastic modulus with the increase of size was also observed for pea [15]. It has been reported that the size of pea influenced its elastic modulus significantly.

### 3.2. Variety

The variation of elastic modulus at different size categories of the investigated varieties of pumpkin seed and its kernel is shown in Table 1. According to this table, the changes in variety significantly influenced the elastic modulus of seed and kernel (*P* < 0.05). Moreover, the elastic modulus of Gaboor variety was about 1.5-fold of Zaria variety of pumpkin seed. Furthermore, the average elastic modulus of Gaboor variety of kernels was significantly more than Zaria variety of pumpkin seed (around 1.85 times). The differences in elastic modulus between the studied varieties could be the result of the individual cultivars properties and different environmental and growth conditions of cultivars. No reported results for elastic modulus of pumpkin seed were found to compare with the results obtained in this study. However, in agreement with these results, a few results can be found in the literature for some grains with some way similarity. For instance, Kiani et al. [18] found that the elastic modulus of red bean varied with variety. They reported the average values of 177.3 and 160.2 MPa for the Goli and Akhtar variety of red bean, respectively. In addition, Khodabakhsina et al. [24] reported that variety has a significant influence on the mechanical properties of sunflower seed and its kernel.

### 3.3. Moisture Content

The elastic modulus of pumpkin seed and its kernel at different moisture contents and size categories is shown in Table 2. As it can be seen, rising moisture from 4% to 20% d.b. showed a decreasing trend in the elastic modulus for both seed and kernel in the cases of size category. In fact, at higher moisture content, the seeds become softer and demand less force. Also, the trend of decreasing elastic modulus at higher moisture contents of kernel may be attributed to a gradual change in the integrity of the cellular matrix. These conclusions are consistent with the findings of Kiani et al. [18] and Burubai et al. [17] who reported that elastic modulus of red bean and African nutmeg decreased linearly with the increase of moisture content. Burubai et al. [17] reported that the modulus of elasticity of African nutmeg was observed to decrease from an average value of 201.5 to 41.30 Mpa, as moisture content increased from 8 to 28.7% (d.b.). These results also agree with the results of Misra and Young [10]. They reported a functional relationship between the modulus of elasticity and moisture content of soybean. They reported that the modulus of elasticity decreased and approached a constant minimum, with the increase in moisture content of soybean. In addition, the results of this section are in agreement with the findings of many researchers who considered the effect of moisture content on mechanical properties of biological products [24, 25, 27–32]. According to Table 2, the maximum elastic modulus of pumpkin seed was 117.48 Mpa, at 4% moisture content and large size. This is significantly more than the elastic modulus of seed at 20% moisture content of the same size (around 1.32 times). The same proportion was observed for the corresponding kernel (around 1.56).

### 3.4. Loading Rate

The effect of loading rate on modulus of elasticity was determined for four loading rates namely 2, 5, 8, and 10 mm/min. The elastic modulus of pumpkin seed and also its kernel decreased as the loading rate increased (Table 3). Burubai et al. [17] observed a negative trend for elastic modulus of African nutmeg with the loading rate. They reported the average value of 135.51 and 120.46 Mpa at 1 and 7 mm/min, respectively. However, Kiani et al. [18] observed that elastic modulus of red bean grain increased with increasing loading rate from 3 to 15 mm/min, for two varieties named Goli and Akhtar. The discrepancies in observed behaviors could be related to the differences in surface roughness of grains or seeds. Investigating the interaction effect of loading rate, moisture content, and variety on modulus of elasticity of both seed and kernel of pumpkin (Table 3) showed that the most differences between varieties belong to Gaboor variety at 4% moisture content and 2 mm/min loading rate. Also, it is denoted that the least differences between varieties belong to Zaria variety at 20% moisture content and 10 mm/min loading rate. In general, the mean values indicate that loading rate, variety, and moisture content produce a significant effect on elastic modulus of both seed and kernel (*P* < 0.05).

The multi variables regression showed that the elastic modulus of pumpkin (both seed and kernel) can be strongly correlated to variables under study (variety, moisture content, loading rate and size category). The results are shown in Table 4. As it is seen, both relationships had high enough coefficient of determination that can be beneficial in estimating modulus of elasticity for the design purpose of processing machineries such as dehulling of pumpkin seed and oil extraction from its kernel.

## 4. Conclusion

Statistically, variation in moisture content, size, and loading rate as well as pumpkin variety (Zaria and Gaboor) either individually or in combination (interaction) was significantly influenced by the modulus of elasticity (*P* < 0.01). In general, negative correlations were observed between elastic modulus of pumpkin seed and also its kernel and moisture content as well as loading rate. In other words, their elastic modulus decreased with increasing moisture content from 4% to 20% d.b. and also decreased with the increase of loading rate from 2 to 10 mm/min for all studied varieties and size categories. On the other hand, a direct correlation was observed between elastic modulus of pumpkin seed and also its kernel with size category from small to large. However, the modulus of elasticity of seeds was significantly higher than that of kernels in all levels of moisture content, variety, loading rate, and size category.

## Figures and Tables

**Table 1:** Mean comparison of elastic modulus (Mpa) of pumpkin seed and kernel considering the interaction effect of variety and size.

Product	Size	Variety	
		Zaria	Gaboor
Seed	Large	88.455a	116.735d
	Medium	54.911b	83.191e
	Small	27.534c	55.814f

Kernel	Large	64.118a	100.835d
	Medium	38.287b	67.288e
	Small	7.761c	35.98f

The means with the same letter is not significant at 5% level according to Duncan's multiple ranges test.

**Table 2:** Mean comparison of elastic modulus (Mpa) of pumpkin seed and its kernel considering interaction effect of moisture content and size.

Product	Moisture content (%)	Size category		
		Large	Medium	Small
Seed	4	117.48a	81.01b	50.49c
	7.8	105.74d	73.43e	44.09f
	14	97.99g	64.24h	36.42i
	20	89.16j	57.51k	35.69i

Kernel	4	101.58a	65.11b	32.65c
	7.8	89.84d	57.53e	25.76f
	14	73.26g	48.34h	17.84i
	20	65.21j	40.17k	11.23l

The means with the same letter is not significant at 5% level according to Duncan's multiple ranges test.

**Table 3:** Mean comparison of elastic modulus (Mpa) of pumpkin seed and its kernel considering interaction effect of loading rate, variety, and moisture content.

Variety	Moisture content (%)	Loading rate (mm/min)							
		2		5		8		10	
		Seed	Kernel	Seed	Kernel	Seed	Kernel	Seed	Kernel
Zaria	4	73.55a	56.38n	70.35e	53.29r	66.99i	49.61t	64.53k	47.37v
	7.8	63.66b	45.83o	60.91f	43.51o	59.08f	42.11o	57.47l	40.27w
	14	56.68c	38.52p	53.35g	35.9s	50.79j	34.3s	47.48m	32.04x
	20	50.28d	30.71q	47.9h	29.06q	45.39h	27.11u	43.04h	27.71u

Gaboor	4	101.83a	85.93o	98.63e	82.73s	95.27i	79.37w	92.80l	76.91z
	7.8	91.95b	76.05p	89.19f	73.29t	87.36j	71.46t	85.75j	69.11t
	14	84.96c	67.81q	81.63g	64.42v	79.07g	62.28x	75.76m	59.55*μ*
	20	78.56d	58.37r	76.2h	56.02r	73.67k	53.66y	71.32n	51.41*α*

The means with the same letter is not significant at 5% level according to Duncan's multiple ranges test.

**Table 4:** The modulus of elasticity (*E*) of pumpkin seed and its kernel as a function of variety (*V*), moisture content (*M*), loading rate (*L*), and size category (*S*).

Product	Relationship	*R* ^2^
Seed	*E* = 114.89 + 28.28*V* − 7.48*M* − 30.46*S* − 2.63*L* − 2.96*V* ^2^ − 0.96*M* ^2^ + 3.25*S* ^2^ + 0.06*L* ^2^ − 0.028*VM* − 0.082*VS* − 0.24*MS* −0.025*VL* + 0.018*ML* + 0.75*SL* + 0.022*VM* *S* + 0.022*VM* *L* + 0.015*MS* *L* + 0.005*VS* *L* 0.006*VM* *SL*	0.98

Kernel	*E* = 89.94 + 31.31*V* − 8.86*M* − 30.30*S* − 0.71*L* − 1.02*V* ^2^ − 1.12*M* ^2^ + 2.81*S* ^2^ + 0.02*L* ^2^ − 0.013*VM* − 0.012*VS* − 0.05*MS* −0.001*VL* + 0.008*ML* + 0.24*SL* + 0.012*VM* *S* + 0.002*VM* *L* + 0.003*MS* *L* + 0.011*VS* *L* 0.008*VM* *SL*	0.98

*R* ^2^: determination coefficient.

## References

[1] Caili F, Huan S, Quanhong L (2006). A review on pharmacological activities and utilization technologies of pumpkin. *Plant Foods for Human Nutrition*.

[2] Stevenson DG, Eller FJ, Wang L, Jane JL, Wang T, Inglett GE (2007). Oil and tocopherol content and composition of pumpkin seed oil in 12 cultivars. *Journal of Agricultural and Food Chemistry*.

[3] Levin JH, Hall CW, Deshmukh AP (1959). Physical Treatments and cracking of sweet cherries. *Michigan Agricultural Experiment Station Quarterly Bulletin*.

[4] Huff ER (1967). Mechanical properties of potato-like Rubber or like Glass. *Maine Farm Research*.

[5] Shelef L, Mohsenin NN (1967). Evaluation of the modulus of elasticity of wheat grains. *Cereal Chemical*.

[6] Fridley RB, Bradley RA, Rumsey JW, Adrian PA (1968). Some aspects of elastic behavior of selected fruits. *Transactions of the American Society of Agricultural Engineers*.

[7] Shelef L, Mohsenin NN (1969). Effect of moisture content on mechanical properties of shelled corn. *Cereal Chemical*.

[8] Arnold PC, Robert AW (1969). Fundamental aspects of load-deformation behavior of wheat grains. *Transactions of the American Society of Agricultural Engineers*.

[9] Arnold PC, Mohsenin NN (1971). Proposed techniques for axial compression tests on intact agricultural products of convex shape. *Transactions of the American Society of Agricultural Engineers*.

[10] Misra RN, Young JH (1980). A model for predicting the effect of moisture content on the modulus of elasticity of soybeans. *Transactions of the American Society of Agricultural Engineers*.

[11] Balastreire LA, Herum FL, Stevens KK, Blaisdell JL (1982). Fracture of corn endosperm in bending: part 1. Fracture parameters. *Transactions of the American Society of Agricultural Engineers*.

[12] Jindal VK, Techasena O (1985). Compression test for measuring the firmness of potatoes. *Transactions of the American Society of Agricultural Engineers*.

[13] Bargale PC, Irudayaraj JM, Marquis B (1994). Some mechanical properties and stress relaxation characteristics of lentils. *Canadian Agricultural Engineering*.

[14] Bargale PC, Irudayaraj JM (1995). Mechanical strength and reological behavior of barely kernels. *International Journal of Food Science & Technology*.

[15] Khazaei J (2002). *Determination of force required to pea pod harvestingand mechanical resistance to impact*.

[16] Hicsasmaz Z, Rizvi SSH (2005). Effect of size and shape on modulus of deformability. *Food Science and Technology*.

[17] Burubai W, Amula E, Davies RM, Etekpe GWW, Daworiye SP (2008). Determination of Poisson’s ratio and elastic modulus of African nutmeg (*Monodora myristica*). *International Agrophysics*.

[18] Kiani M, Maghsoudi H, Minaei S (2009). Determination of Poisson’s ratio and Young’s modulus of red bean grains. *Journal of Food Process Engineering*.

[19] Mohsenin NN (1986). *Physical Properties of Plant and Animal Materials*.

[20] Gupta RK, Das SK (1997). Physical properties of sunflower seeds. *Journal of Agricultural Engineering Research*.

[21] Singh KK, Goswami TK (1996). Physical properties of cumin seed. *Journal of Agricultural Engineering Research*.

[22] Baryeh EA (2002). Physical properties of millet. *Journal of Food Engineering*.

[23] Coşkuner Y, Karababa E (2007). Some physical properties of flaxseed (Linum usitatissimum L.). *Journal of Food Engineering*.

[24] Khodabakhshian R, Emadi B, Abbaspour Fard MH (2010). Some engineering properties of sunflower seed and its kernel. *Journal of Agricultural Science and Technology*.

[25] Joshi DC (1993). *Mechanical dehulling of pumpkin seed*.

[26] ASABE Standards Compression test of food materials of convex shape.

[27] Liu M, Haghighi K, Stroshine RL, Ting EC (1990). Mechanical properties of the soybean cotyledon and failure strength of soybean kernels. *Transactions of the American Society of Agricultural Engineers*.

[28] Bay APM, Bourne MC, Taylor AG (1996). Effect of moisture content on compressive strength of whole snap bean (Phaseolus vulgaris L.) seeds and separated cotyledons. *International Journal of Food Science and Technology*.

[29] Singh KK, Goswami TK (1998). Mechanical properties of cumin seed under compressive loading. *Journal of Agricultural Engineering Research*.

[30] Konak M, Çarman K, Aydin C (2002). Physical properties of chick pea seeds. *Biosystems Engineering*.

[31] Altuntaş E, Yildiz M (2007). Effect of moisture content on some physical and mechanical properties of faba bean (Vicia faba L.) grains. *Journal of Food Engineering*.

[32] Saiedirad MH, Tabatabaeefar A, Borghei A, Mirsalehi M, Badii F, Varnamkhasti MG (2008). Effect of moisture content, seed size, loading rate and seed orientation on force and energy required for fracturing cumin seed under quasi-static loading. *Journal of Food Engineering*.

